# Harmful metals concentration in sediments and fishes of biologically important estuary, Bay of Bengal

**DOI:** 10.1186/2052-336X-11-33

**Published:** 2013-12-19

**Authors:** Shanmugaasokan Lakshmanasenthil, Thirumalairaj Vinothkumar, Thipramalai Thankappan AjithKumar, Thangapandi Marudhupandi, Dhaneesh Kottila Veettil, Raghunathan Ganeshamurthy, Swagat Ghosh, Thangavel Balasubramanian

**Affiliations:** 1 CMS College of Science and Commerce, Chinnavedampati (PO), Coimbatore, Tamil Nadu, India; 2 Centre of Advanced Study in Marine Biology, Faculty of Marine Sciences, Annamalai University, Parangipettai 608 502, Tamil Nadu, India

**Keywords:** Environment, Estuary, Fishes, Health, Metal, Accumulation

## Abstract

Study on the accumulation level of heavy metals was conducted on sediment and fishes from estuaries of Bay of Bengal. Heavy metals were determined by using Inductively Coupled Plasma Optical Emission Spectrometer (ICP-OES) and the results were compared to permissible limits of WHO/USEPA. The accumulation patterns of Fe and Cd were found predominantly in all samples tested when correlated with other metals. It was found that the concentration of metals such as Cd (3.90 ± 0.25 μg/g), Cr (0.44 ± 0.05 μg/g), Ni (0.33 ± 0.01 μg/g), and Mn (1.1 ± 0.11 μg/g) were exceeding the permissible limit, whereas Fe, Co, Pb, and Zn were found within the limit of WHO/USEPA at station 1. In station 2, Cd (16.5 ± 0.4 μg/g), Mn (0.67 ± 0.11 μg/g), and Cr (0.80 ± 0.01 μg/g) were exceeding the permissible limit, whereas Fe, Co, Pb, Ni, and Zn were found within the limit. This study emphasizes that Cd and Mn levels in both stations, are far higher than the acceptable values set by WHO/USEPA and may therefore present human health hazards. It is therefore mandatory to carry out extensive research to evaluate the possible environmental risk factors in the vicinity of both estuaries with respect to heavy metals.

## Introduction

Estuarine ecosystems are highly productive because of rich nutrients received from the river runoff. One of the important roles of the estuaries is to provide the nursery ground for many young marine animals, and thus support a unique community of plants and animals, especially adapted for life in the coastal areas.

The major environmental problem of estuarine ecosystems is receiving various kinds of contaminants from the land. Majority of the industries at coastal area discharge the chemical effluents into the aquatic environment which in turn cause changes in habitat, species distribution, abundance, and bio-geo chemical cycles [1]. Wastes from urban, industries, and mining are the potential sources of heavy metal pollution [2]. The distribution of heavy metals in natural water and water bodies has widely been recognized as a major factor for biological risks [3,4]. As the spawning and nursery grounds of many marine organisms, including commercially valuable shrimp and fishes, which are plenty available in estuarine and coastal areas, they are directly affected by such influx of chemical contaminant into the ecosystem [5].

Fishes are on the top of the aquatic food chain and accumulate large amounts of metals from water and sediment. Sediments are known to be “Trace element traps” [6], because they eventually bind almost all the contaminants which enter the aquatic environment [4]. Heavy metals in sediments can lead to greater environmental problems when the contaminated sediments are resuspended and such metals are taken-up by filter feeders. Sediments can reflect the quality of the water system and can be used to detect insoluble contaminants [4]. Studies from field and laboratory experiments show that accumulation of heavy metals in tissues of animals depends mainly on metal concentration present in water.

Heavy metals normally occurring in nature are not harmful to the environment, because they play an essential role in tissue metabolism and growth of plants and animals [7]. Metals like Cu, Zn, Fe, Co, Mo, Ni, Si, and Sn become predominantly toxic when their level exceeds the limit, and V, Cd, Pb, and Hg are prominently classified as toxic because of their detrimental effect even at low concentrations [8].

The estuaries selected for the present study are biologically important and economic hub of the people and expected to be polluted by industries and human activities. This leads to damage of food chain of this environment by those pollutants are toxic to aquatic animals and human those are live nearby area. Comprehensive studies related to the analyses of sediment and fishes around particular estuaries are limited. Therefore, the present study has been undertaken (i) to evaluate the extent of heavy metal contamination in sediment and edible part of fishes, (ii) to investigate the uptake of eight heavy metals in fishes and (iii) to understand the present condition of the ecosystems and to compile the baseline data for future monitoring.

## Methods

### Study area

Two stations (estuaries) lay in the Bay of Bengal (latitude of 10°55'N; longtitude 79°52'E; altitude of 8.85 m) were selected and the coast has extensive areas of estuaries, mangroves, and brackish water lagoon which all have connection to the Bay of Bengal.

### Sample collection

Two sampling sites (site 1 and site 2) were chosen in both stations. Site 1 (Stacknend), 2 km away from the mouth of the estuary and site 2 (Bar mouth) at the mouth of the estuary.

### Fish

Fifteen fish samples representing each kind of fish species were collected from two stations. Fish species selected for analysis were *Mugil cephalus, Oreochromis mossambicus*, *Arius caelatus, Lutjanus fulviflamma, Terapon jarbua* and *Chanos chanos.* These are commercially and nutritionally important fish species. The samples were collected in sterile sample containers and kept in the laboratory deep freezer (−20°C) to prevent deterioration till further analysis. Total length of fishes was measured and no significant differences were found among the species (*P* > 0.05) (Table 1). For each species, five representative samples with similar lengths were taken for the study.

**Table 1:** Fishes within feasible size range were used for the determination of metals

**S. no.**	**Name of the species**	**Size range (cm)**	**Mode of life**	**Feeding behavior**
1	*Mugil cephalus*	14.5-15.2	Benthopelagic	Heterotroph
2	*Oreochromis mossambicus*	10.3-10.8	Benthopelagic	Omnivores
3	*Arius caelatus*	11.4-11.6	Pelagic	Detrivore and carnivore
4	*Chanos chanos*	25.0-26.4	Epipelagic	Carnivore
5	*Lutjanus fulviflamma*	28.0-29.2	Epipelagic	Carnivore
6	*Terapon jarbua*	34.2-36.0	Epipelagic	Carnivore

### Sediment

Sediment samples were collected at about 30 cm deep from sediment surface to obtain layer of the sediment according to Norhayati et al., (2007) [9]. Triplicate samples were collected from each site for the accuracy. Sampling tools were washed and dried with de-ionized water between the process of sampling, and the collected samples were stored at 4°C.

## Determination of metal concentration

### Fish and sediment

Nitric–perchloric acid digestion method was followed for analysis of heavy metal in sediment and edible tissue of fishes [10,11]. The fishes were descaled and dried at 80°C in an electric oven (Samsung) for 36 h. It was then pulverized to powder using a dry homogenizer and stored. About 1 g (± 0.001 g) of powdered sample was weighed and digested in 100 ml glass beaker containing concentrated nitric acid (20 ml) overnight. It was then mixed with 10 ml of concentrated nitric acid and perchloric acid (4:1) solution. The samples were then subjected to complete dryness by placing on a hot plate. The ash thus obtained was made up to 20 ml solution using ultrapure water from Milli-Q® water system with 20% nitric acid. The mixture was filtered using Whatman filter paper (11 μm) and then the metal concentrations were determined using Inductively Coupled Plasma Optical Emission Spectromter (ICP-OES) (Software- WinLab 32; Perkin Elmer, Optima 2IOODV). The precision of the analytical procedure was checked by analyzing standard reference materials of commercially available standards (Merck KGCA, 64271 Damstadt, Germany, ICP-Multi element standard solution IV, 23 elements in nitric acid). The operating conditions and the wavelength selected for the analysis of different metals by ICP-OES are given in Tables 2 and 3.

**Table 2:** Operating parameters for the Inductively Coupled Plasma Optical Emission Spectrometer

**Parameters**	**Conditions**
Plasma capacity (W)	1300
Plasma flow rate (L minÀ1)	15
Nebulizer gas flow rate (L minÀ1)	0.8
Sample flow rate (mL minÀ1)	1.5

**Table 3:** Wavelength used for ICP-OES analysis for each metal

**Elements**	**Wave length**
Cd	228.802
Co	228.616
Cu	324.752
Fe	238.204
Mg	285.213
Mn	259.372
Ni	231.604
Pb	220.353
Zn	213.857

Sediment samples were air dried in the laboratory at room temperature, ground in fine mixture using mortar and pestle before sieving less than 2 mm mesh. The nitric-perchioric acid digestion method was also followed for the analysis of the sediment.

### Statistical analysis

All analysis was performed in triplicate and mean, SD values were calculated by SPSS® (v16) software for windows. Bray Curtis similarity index was carried out by Primer 6 (v6).

## Results

In the present study, Fe, Zn, Cd, Mn, Pb, Cr, Ni, and Co concentrations were determined in the fishes and sediments of two estuaries. In station 1, accumulation of Fe was found to be higher followed by Zn, Cd, and Mn while Pb, Cr, Ni, and Co were Below Detectable Level (BDL) (Table 4). At Station 2, Cd was found to be a primary pollutant followed by Fe, Zn, and Mn and Pb, Cr, Ni, and Co are in the BDL (Table 5). Depending upon the overall average mean value of each metal, the metal accumulation was in the order of Fe > Zn > Cd > Mn > Pb > Cr > Ni > Co at Station 1, and in the Station 2 samples were in the order: Cd > Fe > Zn > Mn > Pb > Cr > Ni > Co.

**Table 4:** Metal concentration in the station 1 samples

**Metals**	**Fish samples (μg/g)**						**Sediment samples (μg/g)**		**WHO/EPA standard**
	***M. cephalus *** **(Mean ± SD)**	***O. mossambicus *** **(Mean ± SD)**	***A. caelatus *** **(Mean ± SD)**	***C. chanos *** **(Mean ± SD)**	***L. fulviflamma *** **(Mean ± SD)**	***T. jarbua *** **(Mean ± SD)**	**Sst1 (Mean ± SD)**	**Sst 2 (Mean ± SD)**	
Cd	0.13 ± 0.049	2.13 ± 0.368	0.02 ± 0.01	0.23 ± 0.09	0.06 ± 0.02	0.1 ± 0.27	3.9 ± 0.25	1.5 ± 0.27	0.0-0.2
Co	0.008 ± 0.002	0.04 ± 0.004	0.12 ± 0.05	0.02 ± 0.001	0.17 ± 0.02	0.05 ± 0.003	0.03 ± 0.006	0.03 ± 0.01	-
Cr	0.13 ± 0.05	0.11 ± 0.003	0.13 ± 0.04	0.11 ± 0.004	0.1 ± 0.07	0.12 ± 0.07	0.34 ± 0.08	0.44 ± 0.05	0.1-0.15
Fe	14.9 ± 0.25	19.97 ± 0.54	10.3 ± 0.24	3.8 ± 0.16	13.2 ± 1.7	5.2 ± 0.9	12.1 ± 0.4	9.0 ± 0.14	4-48.0
Mn	0.9 ± 0.14	1.2 ± 0.34	0.3 ± 0.04	0.3 ± 0.07	0.1 ± 0.05	0.03 ± 0.01	1.1 ± 0.11	0.2 ± 0.02	0.0025-0.005
Ni	0.31 ± 0.03	0.33 ± 0.01	0.04 ± 0.04	0.05 ± 0.002	0.1 ± 0.03	0.1 ± 0.07	0.06 ± 0.015	0.05 ± 0.034	0.0-0.140
Pb	0.10 ± 0.08	0.13 ± 0.001	0.05 ± 0.03	0.08 ± 0.02	0.25 ± 0.2	0.4 ± 0.05	0.185 ± 0.08	0.0536 ± 0.005	0-1.5
Zn	2.25 ± 0.10	2.26 ± 0.34	2.01 ± 0.45	1.24 ± 0.12	7.1 ± 0.9	1.6 ± 0.8	2.28 ± 0.02	2.9 ± 0.13	58-150

Sst1 *Sediment site 1*; Sst2 *Sediment site 2.*

**Table 5:** Metal concentration in the station 2 samples

**Metals**	**Fish samples (μg/g)**						**Sediment samples (μg/g)**		**WHO/EPA standard**
	***M. cephalus *** **(Mean ± SD)**	***O. mossambicus *** **(Mean ± SD)**	***A. caelatus *** **(Mean ± SD)**	***C. chanos *** **(Mean ± SD)**	***L. fulviflamma *** **(Mean ± SD)**	***T. jarbua *** **(Mean ± SD)**	**Sst1 (Mean ± SD)**	**Sst 2 (Mean ± SD)**	
Cd	16.5 ± 0.4	26.25 ± 0.06	6.95 ± 0.21	14.4 ± 0.197	3.31 ± 0.5	0.9 ± 0.4	1.12 ± 0.01	1.83 ± 0.06	0.0-0.2
Co	0.07 ± 0.002	0.05 ± 0.004	0.01 ± 0.001	0.02 ± 0.003	0.04 ± 0.02	0.03 ± 0.02	0.02 ± 0.001	0.03 ± 0.01	-
Cr	0.06 ± 0.005	0.09 ± 0.006	0.16 ± 0.04	0.8 ± 0.014	0.31 ± 0.1	0.22 ± 0.05	0.17 ± 0.008	0.19 ± 0.07	0.1-0.15
Fe	1.5 ± 0.053	3.9 ± 0.034	3.2 ± 0.05	3.5 ± 0.01	5.7 ± 0.9	6.2 ± 2.1	26.6 ± 2.6	12.9 ± 1.2	4-48.0
Mn	0.18 ± 0.003	0.67 ± 0.11	0.8 ± 0.03	0.6 ± 0.01	0.4 ± 0.3	0.2 ± 0.1	0.5 ± 0.16	0.42 ± 0.03	0.0025-0.005
Ni	0.02 ± 0.002	0.04 ± 0.004	0.03 ± 0.003	0.03 ± 0.002	0.06 ± 0.09	0.05 ± 0.03	0.06 ± 0.01	0.13 ± 0.01	0.0-0.140
Pb	0.05 ± 0.003	0.08 ± 0.005	0.09 ± 0.003	0.06 ± 0.003	0.4 ± 0.09	0.07 ± 0.01	0.8 ± 0.16	0.5 ± 0.36	0-1.5
Zn	1.33 ± 0.04	4.15 ± 0.073	2.4 ± 0.121	2.04 ± 0.10	2.7 ± 0.31	1.7 ± 0.6	3.0 ± 0.20	2.15 ± 0.43	58-150

Sst1 *Sediment site 1*; Sst2 *Sediment site 2.*

The metal concentration and the corresponding mean standard deviations all the samples from both estuaries are shown in Tables 4 and 5. Concentration of the heavy metals significantly varied in the samples collected from the two stations. In station 1, the concentration of Cd ranged from 0.02 ± 0.01 (*A. caelatus*) to 2.1 ± 0.3 (*O. mossambicus*) μg/g, for Co 0.008 ± 0.062 (*M. cephalus*) to 0.17 ± 0.2 (*L. fulviflamma*) μg/g, for Cr 0.1 ± 0.07 (*L. fulviflamma*) to 0.44 ± 0.53 (sst 2) μg/g, for Fe 3.8 ± 0.16 (*C. chanos*) to 19.8 ± 0.58 (*O. mossambicus*) μg/g, for Mn 0.20 ± 0.02 (sst 2) to 1.2 ± 0.34 (*L. fulviflamma*) μg/g, for Ni 0.03 ± 0.01 (*A. caelatus*) to 0.33 ± 0.40 (*O. mossambicus*) μg/g, for Pb 0.05 ± 0.03 (*A. caelatus*) to 1.94 ± 0.01 (sst 2) μg/g, and for Zn 1.24 ± 0.12 (*C. chanos*) to 7.1 ± 0.9 (*L. fulviflamma*) μg/g.

In station 2, the concentration of Cd ranged from 1.12 ± 0.012 (sst 1) to 26.25 ± 0.063 (*O. mossambicus*) μg/g, for Co 0.006 ± 0.003 (*C. chanos*) to 0.049 ± 0.05 (*O. mossambicus*) μg/g, for Cr 0.055 ± 0.005 (*M. cephalus*) to 0.21 ± 0.04 (*C. chanos*) μg/g, for Fe 3.171 ± 0.05 (*A. caelatus*) to 26.58 ± 2.6 (sst 1) μg/g, for Mn 0.42 ± 0.037 (sst 2) to 1.38 ± 0.28 (*A. caelatus*) μg/g, for Ni 0.06 ± 0.01 (sst 1) to 0.13 ± 0.012 (sst 2) μg/g, for Pb 0.05 ± 0.003 (*M. cephalus*) to 0.78 ± 0.16 (sst 1) μg/g, and for Zn 1.319 ± 0.04 (*M. cephalus*) to 4.152 ± 0.073 (*O. mossambicus*) μg/g.

Metals like Fe, Zn, and Mn were commonly found in all the fish samples, but Cd showed the maximum accumulation in Station 2. In case of sediment, there is no much variation in metal accumulation, but Fe, Zn, and Cd relatively showed maximum concentration at both Stations. But in station 2, Cd concentration in fishes was higher than the sediment samples.

**Figure 1:**
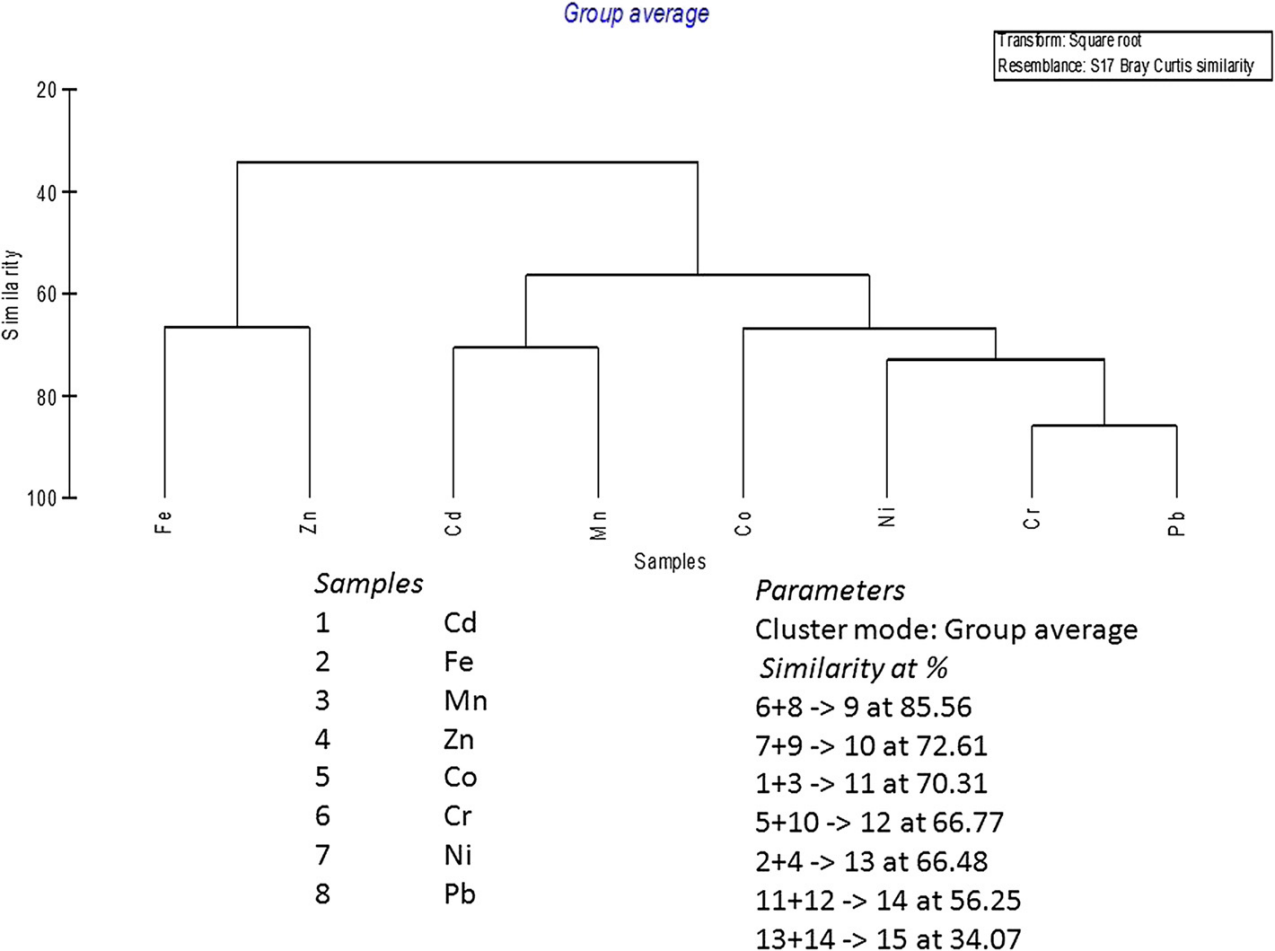
Bray Curtis similarity index versus metals station 1.

**Figure 2:**
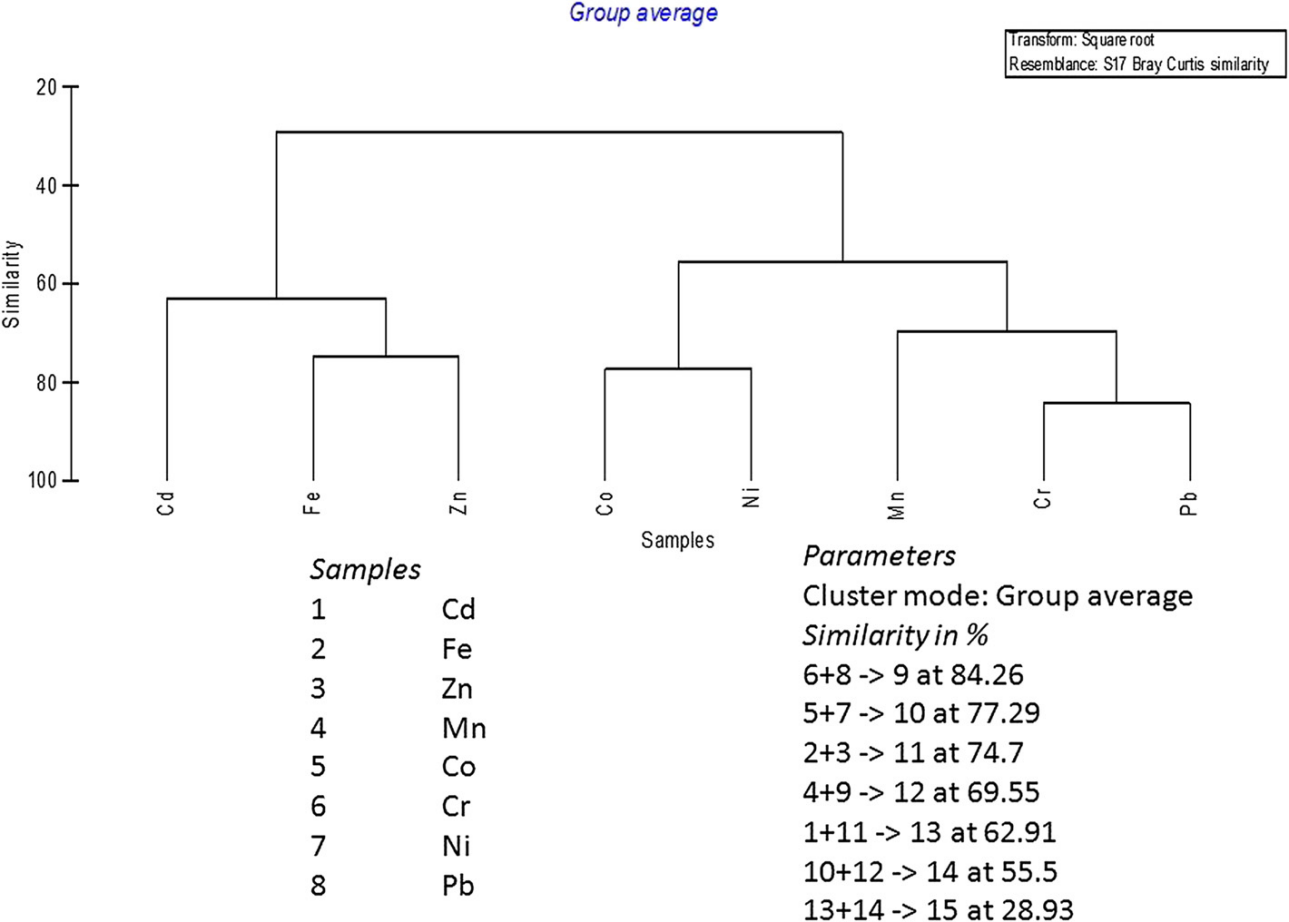
Bray Curtis similarity index versus metals in station 2.

Dendrograms (Figures 1 and 2) are drawn to identifying the percentage of pollution by metals at each station using of group linkage clustering technique (Bray Curtis Similarity coefficient of similarity). Cr and Pb pollutions were at the highest level of (85.56%) of similarity at station 1, Cd and Mn were successfully grouped by the next level of similarity (70.31%). At station 2 same Cr and Pb were polluted at the highest level of similarity (84.26%), Co and Ni showed a second level of similarity (77.29%).

## Discussion

Our study reported that the accumulation of Cd, Cr, Ni, and Mn were exceeding the maximum permissible limit (Tables 4 and 5) and the study stations might face high level risk from metal pollution in the future. In aquatic ecosystems, fishes are considered as good representative indicators of the overall system of health, due to their relatively higher position occupied in the food-chain [12]. Marine organisms, including fish, accumulate heavy metals through direct absorption, or via food chain, and pass them to human beings by consumption, causing acute chronic or disorders [13]. Numerous reports describe the accumulation of metal residues in wild marine fish species [14].

From the observations, sediment samples of station 1, the level of Cd and Mn was higher in site 1 compared to site 2, whereas Zn and Pb were higher in site 2 than site 1. Similar distributions of metals were observed in fish samples of both sites of station 2. These results indicated that site 1 (stacknend) was more polluted than site 2 (bar mouth) because all the waste and industrial effluents were entered through site1 (stacknend).

The different forms and concentration of heavy metals in the sediment of an aquatic medium determine their bioavailability and toxicity. Thus, the study of the different fractions of the elements in the sediment was vital because the total concentrations are not representative of the real degree of the potential contamination. Heavy metals can be bound to or occluded in amorphous materials, adsorbed on clay surfaces or iron/manganese oxyhydroxides, co-precipitated in secondary minerals such as carbonates, sulfates, or oxides, complexes with organic matter, or included in the lattice of primary minerals such as silicates [15].

In the current study, heavy metal concentrations in sediment were found to be higher in for fishes at both stations. As such, accumulation of heavy metals in tissue depends mainly on metal concentration in sediment. This shows that Stacknend station was more polluted than the Barmouth. The sediment samples also reflected the same trend.

Metal Pollution Index (MPI) (safe limits) of WHO distinguishes polluted from unpolluted ecosystems, based on acquired knowledge on metal bio-availability, bio-concentration and bioaccumulation patterns [16]. Results of our study shows, fish samples such as *M. cephalus, O. mossambicus, A. caelatus,* and *C. chanos*, *T. jarbua* and *L. fulviflamma* collected from station 1, Fe and Zn accumulated highest concentration, whereas Co and Pb had the lowest among all eight metals analyzed. But based on WHO/USEPA reference standard for fish [17] (Table 4), Cd, Mn and Ni were the major pollutant in station 1. In *O. mossambicus* and *C. chanos,* the Cd (2.128 and 0.238 μg/g) was found higher than the standard value of WHO/USEPA. But Mn (0.859, 1.252, 0.283 and 0.276 μg/g) and Ni (0.314, 0.327, 0.038 and 0.036 μg/g) were found to be in an elevated state (concentration) than the standard value in all fish samples of Station 1. The levels of Fe, Zn, Pb, Cr, and Co were found were found to fall within WHO/EPA safe limits. Fish samples collected from Station 2, concentration of Cd was higher than other metals and Co was at the lowest concentration. These results are indicative that Cd (16.69, 46.3, 6.94 and 14.37 μg/g), Mn (0.181, 0.698, 0.794 and 0.642 μg/g), and Cr (0.155 μg/g) were relatively higher than the standard value (Table 5).

Metals like Fe, Cu, Zn, and Mn are the essential metals for life, whereas Ni, Pb, Co, and Cd are toxic even in trace amounts. Lakshmanasenthil et al., (2012) [18] reported that the Cd accumulation (1.11, 3.85 and 2.53 μg/g) in different organs of same species from Nagoor estuary, Bay of Bengal. It has been shown that that Cd was the major pollutant of that area and that it is has been proved that Cd accumulation in fishes will lead to their liver damage.

Vinodhini and Narayanan (2008) [19] also proved the high concentration of Cd (1.883, 1.693 and 1.166 μg/g) alters the metabolic functions of *Cyprinus carpio* fish. Metal contaminants eventually find their way into the human system through the food chain [20], Harvey (1975) [21] reported the death of human who consume Cd-polluted fish from the Jintsu River estuary in Japan. The present research showed that concentration of Cd in the sample is at an alarming level which may affect total environment and cause dangerous consequences.

Harvey (1975) [21] reported that the Mn concentration has been shown to accumulate in the kidney, bone, liver tissues and also induces sexual excitement and impotence of marine fishes. Indrajit et al., (2011) [22] explained the pollution of Mn (5.14, 1.19 μg/g etc.,) in Yamuna river by fishes. Thiyagarajan et al., (2012) [23] showed cadmium, chromium, manganese and mercury concentration of five marine fish species (*Epinephelus chlorostigma, Lutjanus russelli, Terapon jarbua, Cynoglossus arel* and *Lagocephalus lunaris*) varied from 0.274 ± 0.03 to 53.39 ± 14.3 μg/g which led to total environment pollution in Cuddalore, nearest place of our study stations. Nickel is a hazardous element notified by the USFDA (1993) [24], though not covered by EC regulations for fish and other aquatic products. However till now, no exact report has been made on the metal accumulation by the fish species selected for the present study. To the best of our knowledge, this is the first report on the metal accumulation of *Mugil cephalus, Oreochromis mossambicus*, *Arius caelatus, Lutjanus fulviflamma, Terapon jarbua* and *Chanos chanos*.

In our study, the concentrations of Fe, Zn, Pb, and Co of both estuaries were in safe limit. Though these metals are within safe limit at present, there is an increasing chance that it might get increase in the near future, and turn toxic. Cobalt is one of the metal that is present naturally in the body, but in excess concentration, it becomes toxic and lead to harmful potentially permanent side effects like cardiomyopathy, hypothyroidism, and neurological damage as well as impairment of the senses [25]. Similarly, iron poisoning causes pain in the stomach, due to ulceration of stomach lining; which is accompanied by nausea and vomiting. Zinc toxicity is a rare phenomenon but it may induce toxicity when concentration increased to about 40 μg/kg. In addition to this several in vitro studies indicated that high concentrations of chromium (III) in the cell can lead to DNA damage [24]. Chromium reaches the blood stream; it damages the kidneys, the liver, and blood cells through oxidation reactions. Lead is a nonessential toxic element which can cause defects in survival, growth rate, development, and metabolism in fishes [13]. These previous findings suggest that these metals turn toxic to humans when they get accumulated in excess level through food like fishes.

## Conclusion

The results of this study provided valuable information about the metal contents in sediment and fish from the two estuaries of Bay of Bengal. Based on the findings, station 2 was more polluted than station 1 because of the high accumulation of Cd. The marginally higher concentrations of Cd, Cr, and Mn in the samples collected from station 2 could be related to industrialization and related anthropogenic activities in these areas. Moreover, these results enable us to understand the chemical quality of fish and to evaluate the possible risk associated with their consumption. This study emphasizes that Cd and Mn levels in both stations, are higher than the acceptable values for human consumption set by various health organizations and presents a greater human health issue in the future. These results insist that station 2 should be monitored on a regular basis for more possible metal toxicity. Besides, further studies are needed to evaluate and to characterize the fishes in the estuary relative to specific metal pollutants because the Cd level in fishes of station 2 and Fe level in fishes of station 1 were higher compared to sediment. This study is an indication of high contamination of water resource due to the anthropogenic sources and the competent monitoring program is an essential adjunct to any attempt of managing the coastal areas in an ecologically sound and sustainable manner.

## Competing interests

All authors declare that they have no competing interest.

## Authors’ contributions

SL and TM were the main investigator, designed and performed the study and drafted the manuscript. TV and TTA supervised the study. KVD, JG and TB were advisors of the study. SG helped in the statistical analysis. All authors read and approved the final manuscript.
